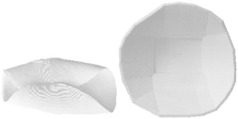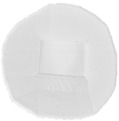# A New Method for Testing the Breaking Force of a Polylactic Acid-Fabric Joint for the Purpose of Making a Protective Garment

**DOI:** 10.3390/ma15103549

**Published:** 2022-05-16

**Authors:** Slavica Bogović, Ana Čorak

**Affiliations:** 1 Department of Clothing Technology, Faculty of Textile Technology, University of Zagreb, 10000 Zagreb, Croatia; 2 Primary School Dr. Franjo Tudjman, 53230 Korenica, Croatia; ana.corak2@skole.hr

**Keywords:** 3D printing, textile applications, polylactic acid (PLA), breaking force, knee protector

## Abstract

3D printing is a technology that is increasingly used in the individualization of clothing, especially in the construction of garments for people with disabilities. The paper presents a study on the use of 3D printed knee protectors intended for wheelchair users. Due to the specific purpose of this 3D printed object, the breaking force of the polylactic acid (PLA) combined with 100% cotton and 100% polyester fabric was investigated. This paper will also describe a new method for testing the breaking force of a 3D printed polymer (PLA) combined with an incorporated fabric. Test samples were made, and the input parameters used in 3D printing were defined for testing purposes. A 3D knee protector for wheelchair users was developed based on a digitized model of the human body. The durability of the shape of the 3D printed shield was also tested after washing at temperatures of 40 °C, 50 °C and 60 °C. A clothing model that provides adequate user protection was proposed based on the conducted research. A construction solution has been proposed that enables the application of a 3D printed individualized garment element.

## 1. Introduction

The application and development of individualized products based on 3D printing have been on the rise in the recent years. In the production of clothing, 3D printing is used to make entire garments, in which cases its segments are not integrated in the garment, or for the printing of patterns or incorporated elements on textile materials [1,2,3,4]. The additional function of the garment can be achieved in terms of design, the functionality of the garment (e.g., protection) or for the purpose of incorporating electronic components into the garment [5,6]. Different 3D printing technologies are used for 3D printing: fused deposition modeling (FDM) printers, stereolithography (SLA) printers, selective laser sintering (SLS) or polyjet modeling (PJM). For all of the above mentioned 3D printing technologies, it is necessary to create a digital 3D model in stereolithography file (STL) format that is converted into G-code, containing all the data needed for 3D printing [7].

Acrylonitrile butadiene styrene (ABS), polyethylene terephthalate (PET), polylactic acid (PLA), nylon and thermoplastic polyurethane (TPU) are polymers most commonly used for the 3D printing of garments, clothing segments and patterns or incorporated elements on textile materials. The choice of polymer depends on the final application of the garment, as well as the properties of the polymer and textile materials. The adhesion of different polymers to textile materials has been investigated in previous studies. The influence of textile surface properties on the adhesion strength of plastic flexible polymers is also investigated, taking into account the mechanical/physical and chemical mechanisms of adhesion using FDM technology [2,7,8,9].

To achieve higher adhesion strength, tests were conducted on the influence of mechanical and thermal parameters of 3D printing, such as the distance of layers from the material along the z-axis [10,11,12], chemical factors [6] and the treatment of textile material with plasma [9,13]. The treatment of textile surfaces with polymeric materials showed an increase in adhesive strength [8,14]. The bond strength of textile material and polymers is examined by varying a large number of 3D printing parameters, taking into account the raw material composition of textile material, the material density and the type of textile fabric [2,15].

Maintenance plays a major role in the functionality of the garment. Therefore, adhesion studies of 3D printed polymers after washing are performed regarding the number of repetitions of the wash cycle. This enables the collection of relevant data that can be used for the correct choice of polymer and textile material regarding its density and structure and their combinations. Based on these studies, it can be determined which combinations of polymers and materials are suitable for long-term or short-term use, as well as the combinations suitable only for prototyping [6,16].

PLA is a polymer that is increasingly used in FDM-based 3D printing due to its properties. It has sufficient mechanical strength, is biodegradable and can be adapted to different purposes by varying the parameters of the 3D print and the 3D modeling process. In its purest form, it has properties that are not suitable for the long-term use of 3D printed objects. PLA polymer composites are used for 3D printing. Such polymer composites directly affect the solubility and mechanical properties of the polymer, as well as the properties of the 3D printed object [17,18].

Clothes for wheelchair users [19] should be made according to individual measures and the possibilities of using these garments to ensure adequate protection. The use of 3D technology can therefore be a key element in ensuring adequate protection. A 3D body scanner is used to digitize the human body, take measurements and define the shape of the human body with all its specifics in a short time. By using the digitized model of the human body, it is possible to make a customized garment and define measures and forms of protective elements for each person specifically [19,20]. Therefore, the possibilities of applying 3D technology can be further explored for the development of individualized clothing for wheelchair users that differs from standard clothing. The shape and properties of the 3D printed protective elements of a garment are also investigated. In order to ensure the functionality of such garments and to facilitate their handling and use, it is necessary to examine the possibility of incorporating textile material into a polymer that is shaped according to the part of the body for which protection is provided [20]. It is necessary to test the strength of the bond of the polymer and textile material in order to fix the 3D printed protection element on the garment, which is shaped and positioned exactly according to the shape of the user’s body.

When it comes to clothes for wheelchair users, it is necessary to develop functional clothing in a studious way in order to enable easier dressing in addition to protection, given the limited movements of the users [20].

A method of testing the breaking force of the bond between the polymer and textile material was developed with that purpose. Past research was based on testing the strength of the bond where the polymer was applied to the textile material. The samples were defined according to the standards that were the foundation for the mentioned research [2,8,9,11,13,15,18,21].

## 2. Materials and Methods

This research on the application of 3D printed polymer PLA for the purpose of making protective elements that are an integral part of clothing was conducted in three steps to find the best 3D print parameters that affect the quality and usability properties of 3D printed individualized knee protectors for wheelchair users.

The breaking force of the bond between the polymer and textile material on the samples was tested on samples where the textile material is located between the layers of 3D printed polymers of different fillings. Knee protectors were made based on a 3D scan of the human body. The stability of the shape of the 3D printed knee protectors of different wall thicknesses [20] was tested at different washing temperatures.

### 2.1. Materials

Biodegradable polymer PLA filament (produced by Devil Design Ryszka Mateja Company, Poland) was used for the experiment. The filament has a diameter of 1.75 mm, a density of 1.24 g/cm^3^, an extrusion temperature of 200–250 °C and a heated bed of 50–60 °C. The basic parameters of 3D printing were defined, as shown in Table 1.

The textile materials for which the tensile strength tests were performed are 100% cotton and 100% polyester. The characteristics are shown in Table 2.

### 2.2. Methods

3D printing is a method of creating objects based on a digital 3D model. The method of 3D printing using FDM technology is based on layering a polymer which is heated and melted at certain temperatures. The melted polymer passes through the nozzle above the bed of the 3D printer. The nozzle can be of different diameters depending on the object and polymer used for 3D printing. It moves along the x and y axes, leaving behind a thin strand of polymer. The polymer is transported toward the nozzle using rollers, which prevents the clogging of the nozzle. After one pass, the mechanism holding the nozzle rises along the z axis. In this way, the model is created layer by layer, attaching itself to the preheated bed in the first pass and to itself in all of the subsequent passes.

The process takes place according to the G-Code generated from a stereolithography file of a 3D object and the printing parameters. The parameters are defined according to the final use of the 3D object and the polymer in use. Depending on the properties of the polymer, it is necessary to define the temperature of the nozzle and bed, as well as the manner in which the polymer is to be fed to the nozzle. Parameters of 3D printing such as wall thickness (number of layers), layer height, object infill (density and shape), support, the speed of printing and others are determined depending on the use of the 3D object. The printing support must be defined for parts of the object that do not have a previous layer on which they could be printed. It is required to construct the 3D digital object shape in a CAD program, taking into account all of the features and limitations of the 3D printing method. 3D construction and G-Code preparations can reduce the need for support, which reduces the time of printing and polymer use.

Based on the described method of 3D printing, samples were made to test the breaking force of the PLA/textile/PLA bonds of samples that were supposed to be incorporated into the garment.

### 2.3. Preparation of the 3D Sample Model

Investigation of the influence of 3D printing parameters with FDM technology on the breaking force of a sample that has an integrated fabric between the polymer layers was performed by adapting the sample to the standards of HRN EN ISO 13934-1: 2013 (Textiles—Tensile properties of fabrics—Part 1: Determination of maximum force and elongation at maximum force using the strip method (ISO 13934-1: 2013; EN ISO 13934-1: 2013)) [22].

**Figure 1:**
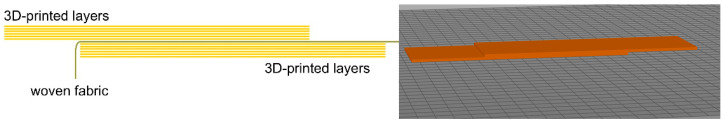
3D sample model for breaking force test.

For the investigation of the breaking force, test samples were made in accordance with the method of testing prescribed by the stated norm. To test the breaking strength of the textile material between the layers of the 3D printed polymer, a 50 × 200 mm sample model was constructed, as shown in Figure 1. The dimensions of the central part of the sample into which the fabric is integrated were 50 × 100 mm. The end sides of the test sample were used to secure the test sample to the dynamometer clamps.

Test samples for measuring the breaking force were prepared using a Creality CR-10 Max 3D printer. The bed of the 3D printer is 450 mm × 450 mm in depth and width and 470 mm in height. Six types of samples were prepared, varying the fill density, as shown in Table 3. The 3D printing of the samples was performed with filling densities of 20%, 60% and 100% for 100% cotton and 100% polyester fabric.

## 3. Results and Discussion

### 3.1. Breaking Force

**Figure 2:**
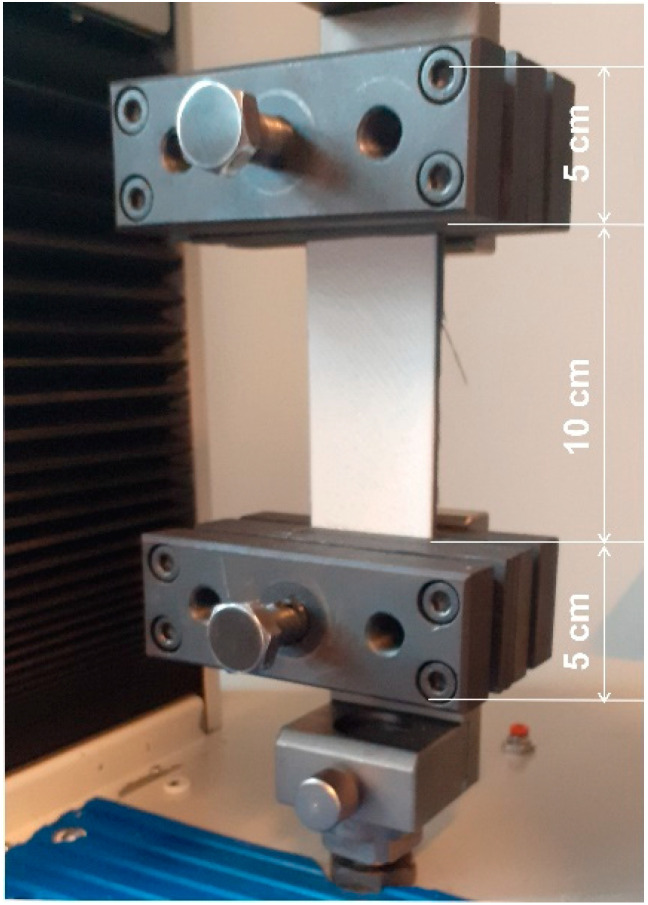
Sample testing on a MesdanLab Strength Tester dynamometer.

The breaking force of the PLA/textile/PLA bond was tested and measured on the prepared samples on a MesdanLab Strength Tester dynamometer. Figure 2 shows a protector sample clamped in the dynamometer. The sample was pre-tensioned with 0.5 N.

**Figure 3:**
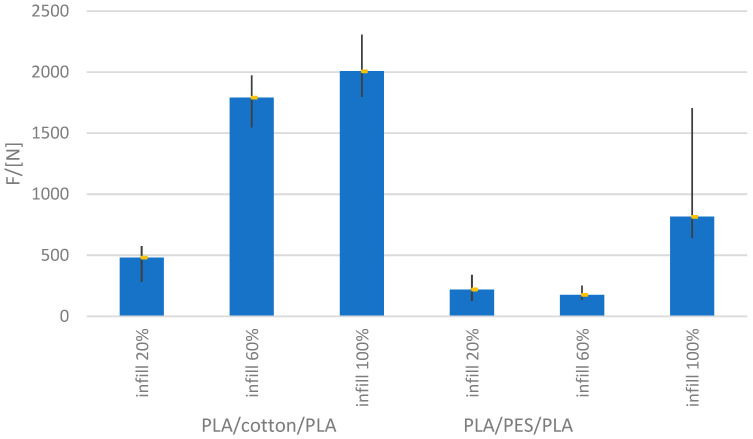
Test results of the breaking strength of PLA/cotton/PLA and PLA/polyester/PLA with fillings of 20, 60 and 100%.

**Figure 4:**
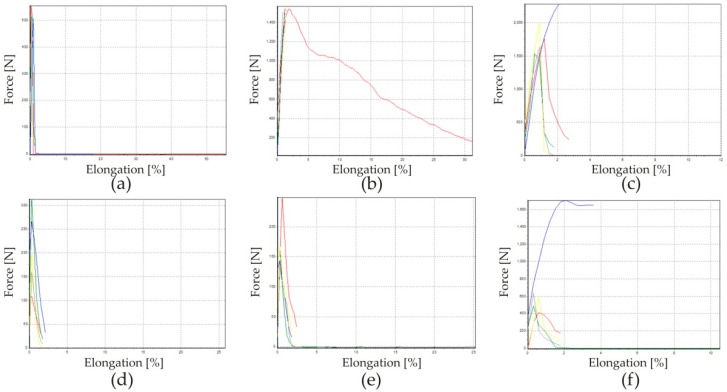
Test results of the elongation of PLA/cotton/PLA with fillings 20% (**a**), 60% (**b**) and 100% (**c**); and PLA/polyester/PLA with fillings 20% (**d**), 60% (**e**) and 100% (**f**).

Based on the prepared samples for testing the breaking strength of the PLA/textile/PLA bond, the results were obtained for fabrics of raw material compositions of 100% cotton and 100% polyester (PES). Different 3D printed samples of 20, 60 and 100% density were used, and the results are shown in Figure 3. Figure 4 shows a graph of the elongation of the samples.

Based on the presented results of measurements performed under the same conditions, it can be observed that there is a deviation in the amount of breaking force depending on the filling density of the 3D printed samples and the type of fabric used. The fabrics used in the test are of approximate density. The deviation in the breaking force values for PLA/cotton/PLA and PLA/polyester/PLA for samples of the same filling density is significant.

According to the obtained results, it can be concluded that the raw material composition of textile material has a significant influence. At the same percentage of filling (60%), the breaking force of the 3D printed element with incorporated cotton material is many times higher than when incorporating polyester fabric. In the case of cotton fabric with 100% filling, the breaking force is twice as high as in the case of the polyester sample at the same filling value. Previous studies on the adhesion of polymers to fabrics have also shown the better adhesion of polymers to cotton materials than polyester, although the polymer was applied only on the upper side of the fabric [21]. The difference between the breaking force in the test of cotton material and fillings of 60 and 100% is not negligible, and the presented results show that the breaking force does not increase linearly. Besides the 3D printing parameters, the sequencing of the layers also has an effect on the breaking force. The sequence is defined by the G-Code and is automatically generated after setting the 3D printing parameters. The 3D printing of higher density objects takes longer, which allows more time for the previous layer to cool down and harden. If textile is placed between two such layers, inconsistency in surface filling occurs.

The results show that there is a discrepancy between the values of the elongation and the breaking force of the same group of samples. The dispersion of data might be caused by the unevenness of the textile material or the unevenness of the 3D printing sample.

During the test of the breaking force, it was noticed that the separation of the test sample occurred between the textile material and the lower layer of the polymer, while the fabric remained adhered to the upper layer of the polymer. Since these are low density fabrics, the polymer has passed through the fabric. The aforementioned facts indicate that there is a need for new discoveries in the application of 3D printed objects onto textile materials. The strength of the bond can be further increased by reducing the fabric density or by using construction methods by defining openings at the PLA/fabric/PLA bonds.

### 3.2. Application onto the Garment

**Figure 5:**
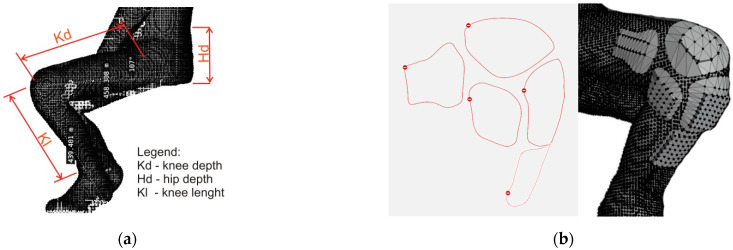
Defining body measures (**a**) and protector shape (**b**) according to the 3D human model.

The stability of the 3D model of the protector was investigated after testing the breaking force of the polymer/fabric/polymer bonds. The 3D model of the protector is based on the 3D model of the human body. The positions and shapes of the protective elements of the garment were determined by analyzing the 3D human model. Point cloud segments of the human body serve as a foundation for 3D shield modeling, while body measurements serve to develop and construct the garment into which the shields are incorporated (Figure 5). The construction of clothing and protectors is carried out in two different ways. The trousers are constructed two-dimensionally, while the protectors are constructed using a 3D modeling software package.

**Figure 6:**
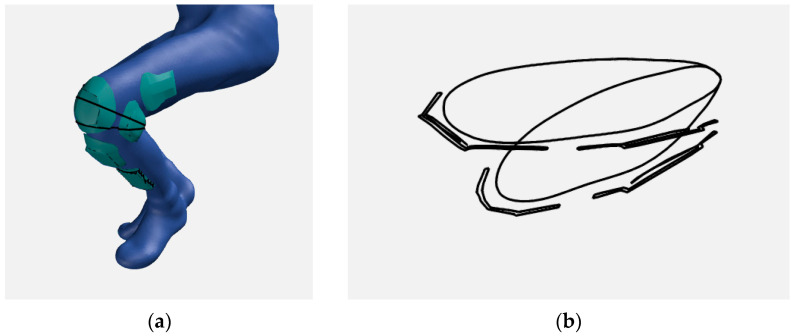
Defining the intersection of the protector and knee (**a**); Defining the comfort and adherence of the 3D printed element and knee (**b**).

**Figure 7:**
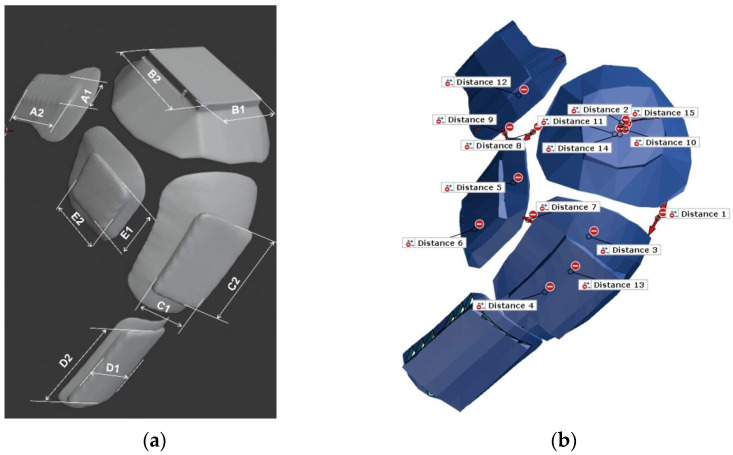
Determining the dimensions of the protector applied to the material (**a**); and the distance between protector elements (**b**).

To create a 3D element that is integrated into a garment, it is necessary to take into account a number of parameters that affect the final shape and function of the garment. The comfort and adherence of the 3D printed element are crucial, because solid protective elements cannot be subsequently adapted to the body shape (Figure 6). Knowledge of 3D printing technology is also very important, because it significantly affects the 3D design of objects. Thus, with FDM 3D print technology, it is important to take into account the polymer layering and the angle of inclination of the walls, wall thickness, etc. Since this is a human body with all its specifics, it is necessary to shape the protective elements according to the body. Parallel to the design of the 3D printed element, it is necessary to carry out the construction of the garment, which is additionally shaped according to the 3D printed element in order to finally connect two different elements and ensure the function for which it is intended (Figure 7). To bond two different materials of polymer and textile material, a regular plate is placed as a base into which the textile material is integrated [20].

**Figure 8:**
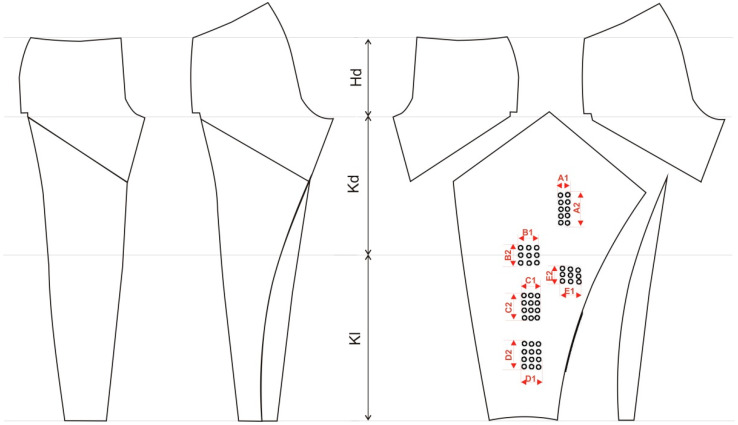
Cutting parts for 3D printing of protectors by incorporating textile material into the polymer.

Since textile materials of higher strength are commonly used in the production of protective garments resulting from the greater thickness and density of the textile material, it is necessary to consider the possibility of applying design solutions that allow for the better incorporation of textile material into 3D printed individualized protective elements. The application of 3D printed shapes in garments can thus be increased. The construction solution shown in Figure 8 is proposed for this purpose. The construction of the garment is adapted to the body in the sitting position. The entire process of garment construction is also adapted to the technical performance of 3D printing.

The body size shown in Figure 4a was used to adapt the cut of the trousers for a person in a sitting position. Measures for knee depth (Kd), hip depth (Hd) and knee length (Kl) were defined, as they have an effect on the fitting of the garment. The measurement also defines the exact positions of the protector incorporated into the garment. The 3D constructed protectors have a rectangular base that allows for easy integration of the fabric. The dimensions of the base, as well as all of the parts of the protector and the distance between the protectors, are shown in Figure 6. The positions of the protectors were exactly calculated based on the above mentioned measurements (Figure 8). This modeled individualized cutting part of the trousers provides the possibility of integrating a flat cutting part into 3D models of the shield.

**Figure 9:**
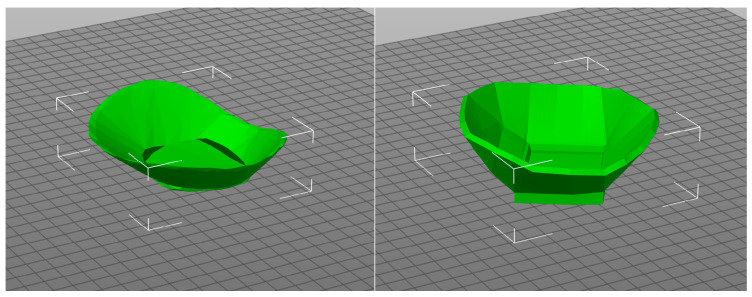
Knee protector made according to a 3D scan of the human body with a wall thickness of 7 and 12 3D printed layers [20].

Two protectors of different wall thicknesses (7 and 12 3D printed layers) were made to investigate the stability of the shape of the protector in the washing process at different temperatures (Figure 9).

Since the combination of the polymer and textile itself is not the only factor that indicates the functionality and usable properties of the finished garment, it is necessary to conduct tests of shape stability. To test the stability of the shape, the protectors were subjected to a washing process at temperatures of 40, 50 and 60 °C. The washing procedure was repeated five times for those elements that did not change shape in the previous wash cycle. During the washing process, the samples were left for 45 min in the aforementioned temperatures.

The samples of a 3D printed element with a wall thickness of seven layers of 3D printed PLA were washed at different washing temperatures (40, 50 and 60 °C), where deformations of the shape are clearly visible. Table 4 shows the results obtained after testing the stability of the shape of a 3D printed object with different wall thicknesses at different washing temperatures.

The protective element with a smaller wall thickness (seven layers) was deformed at washing temperatures of 50 and 60 °C after the first wash cycle, while its shape remained unchanged at a temperature of 40 °C. Therefore, the undeformed protector was subjected to further washing cycles at a temperature of 40 °C.

The thick-walled knee protector retained its shape even after five wash cycles at all of the tested wash temperatures.

After five washing cycles, it was noticed that the surface of the 3D fastened elements became rougher, while the edge became uneven with the shield of smaller wall thickness. The reason for this is the dissolution of the polymer in water, which indicates the reduced durability of the built-in 3D printed elements in the garment. Based on the presented results, it can be concluded that the wall thickness of the 3D printed element affects the stability of the shape in the washing process. This research is necessary because it indicates the applicability of 3D printed elements incorporated into a garment. The optimal wall thickness, in addition to ensuring the stability of the shape in the washing process, will ensure adequate protection of the part of the body for which the protective element is intended.

## 4. Conclusions

Based on the presented results, it can be concluded that there is a need for further research related to the application of 3D printed elements that are incorporated into garments in order to achieve additional functionality that is primarily concerned with protection. Previous research on the adhesion of polymers to textile materials conducted by the method of 3D printing on the material, along with the presented research, indicates the possibility of incorporating textile material into 3D printed polymers by developing a new method of testing the breaking force of the polymer/textile/polymer. By varying the parameters of 3D printing, it is possible to determine the optimal parameters of 3D printing and the type of textile product that will ensure the adequate application of the garment. It is also evident from the above that the construction of clothing and the 3D modeling of the elements integrated into the garment are interdependent and that design solutions can be found to provide adequate and reusable garments, especially for sensitive target groups such as people with disabilities.

## Figures and Tables

**Table 1:** Basic parameters of the 3D print polylactic acid (PLA) used to test the breaking strength of the bond with 100% cotton and 100% polyester fabric.

Filament Data			3D Print Settings					
	Extrusion Temp. [°C]	Diameter [mm]	Speed [mm/s]	Density [g/cm^3^]	Layer Height [mm]	Bed Temp. [°C]	Nozzle Size [mm]	Fill Angle
PLA	210	1.75	30	1.24	0.3	60	0.4	45°

**Table 2:** Characteristics of textile materials bonded with polylactic acid (PLA) for determining the breaking force.

Fabric	Raw Material Composition	Density [Thread/cm]		Weave	
		Warp	Weft		
1	100% cotton	25	24	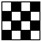	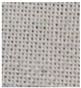
2	100% polyester	18	22	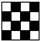	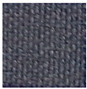

**Table 3:** Defining 3D printed pattern fillings.

Infill Density	Preparation for 3D Printing	3D Printing of Model Infill
20%	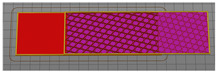	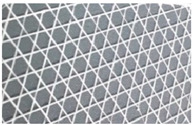
60%	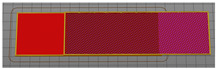	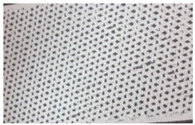
100%	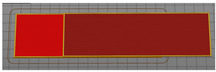	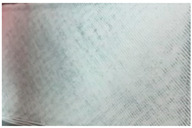

**Table 4:** Results of shape stability testing at different washing temperatures.

Washing Temperature	After a Single Wash	After 5 Washes
40 °C	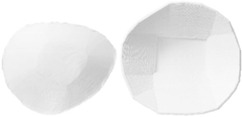	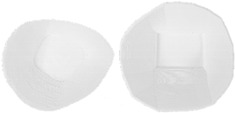
50 °C	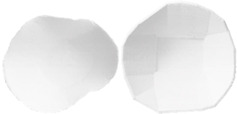	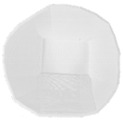
60 °C